# Supplementary material for: Cheminformatics-Based Study Identifies Potential Ebola VP40 Inhibitors
Source: Int J Mol Sci. 2023 Mar 27;24(7):6298. doi: 10.3390/ijms24076298 (PMC10094735; doi:10.3390/ijms24076298)
Supplement: Supplementary file 1 [file ijms-24-06298-s001.zip › ijms-2280369-supplementary.pdf]

Supplementary Information

Content

Table S1: Predicted physicochemical and biological properties of some shortlisted compounds using SwissADME.
Table S2: Anti-EBOV inhibition efficiency of the shortlisted compounds using Anti-Ebola.
Table S3: Interacting residues of the VP40 with nilotinib and the potential lead compounds during molecular dynamics simulations.

Figure S1: Potential energy plot of the VP40 after subjecting the protein to energy minimization using CHARMM-36 and OPLS force fields.
Figure S2: Protein-ligand interaction map of VP40 in complex with: (**a**) ZINC000034518176; (**b**) ZINC000095485942; (**c**) NANPDB2933; (**d**) ZINC000014089759; (**e**) ZINC000085545967; (**f**) ZINC000085568136; (**g**) ZINC000095912717; (**h**) doramectin; (**i**) ledipasvir; and (**j**) abamectin.
Figure S3: RMSF plot of the EBOV VP40-ligand complexes after 100 ns MD simulations.
Figure S4: Hydrogen bond analysis plot of the EBOV VP40-ligand complexes after 100 ns MD simulations.
Figure S5: Per-residue energy decomposition plots of the various complexes showing the energy contributions of each amino acid residue for (a) VP40-nilotinib, (b) VP40-ZINC000034518176, (c) VP40-ZINC000095485942, (d) VP40-ZINC000085531689, (e) VP40-ZINC000014089759, (f) VP40-ZINC000085568136, and (g) VP40-ZINC000095912717 complexes.

Supplementary Tables

Table S1. Predicted physicochemical and biological properties of some shortlisted compounds (the remaining TCM compounds) using SwissADME.

Compound	Binding Energy (kcal/mol)	Molecular Weight (g/mol)	TPSA (Å^2)	LogP	ESOL Solubility Class	GI Absorption	BBB Permeant	Pgp substrate
ZINC000100822646	-8.4	494.49	135.29	4.28	Poorly soluble	Low	No	No
ZINC000100822774	-8.4	494.49	135.29	3.92	Poorly soluble	Low	No	No
ZINC000103580868	-8.4	576.93	18.46	3.92	Poorly soluble	Low	No	No
ZINC000014587019	-8.3	456.49	99.38	4.22	Poorly soluble	High	No	No
ZINC000033829866	-8.3	452.67	43.37	5.93	Poorly soluble	Low	No	No
ZINC000095910238	-8.3	568.87	32.76	4.66	Poorly soluble	Low	No	No
ZINC000103580368	-8.3	526.53	137.96	0.81	Soluble	High	No	Yes
ZINC000004098631	-8.2	440.49	79.15	5.02	Poorly soluble	High	No	No
ZINC000033833171	-8.2	456.7	57.53	5.45	Poorly soluble	High	No	No
ZINC000070454883	-8.2	427.62	58.56	3.54	Moderately soluble	High	Yes	Yes
ZINC000095911414	-8.2	416.42	108.74	3.72	Moderately soluble	High	No	No
ZINC000033833170	-8.1	456.7	57.53	5.48	Poorly soluble	High	No	No
ZINC000070451048	-8.1	530.65	119.49	2.97	Moderately soluble	High	No	No
ZINC000085530490	-8.1	568.65	124.8	3.35	Moderately soluble	High	No	Yes

ZINC000085592933	-8.1	582.64	117.84	4.32	Poorly soluble	Low	No	No
ZINC000095910059	-8.1	532.67	111.52	2.98	Moderately soluble	High	No	Yes
ZINC000095911347	-8.1	526.53	137.96	1.37	Soluble	High	No	Yes
ZINC000095911794	-8.1	436.58	69.92	6.03	Poorly soluble	Low	No	No
ZINC000095912227	-8.1	442.72	40.46	6.2	Poorly soluble	Low	No	No
ZINC000103554058	-8.1	526.53	137.96	3.68	Poorly soluble	High	No	Yes
ZINC000005765422	-8	442.72	40.46	6.22	Poorly soluble	Low	No	No
ZINC000014587014	-8	456.49	99.38	4.18	Poorly soluble	High	No	No
ZINC000014828729	-8	437.53	68.23	3.72	Moderately soluble	High	Yes	Yes
ZINC000031155872	-8	484.5	124.8	2.06	Soluble	High	No	Yes
ZINC000049888703	-8	592.68	72.86	5	Poorly soluble	High	No	No
ZINC000070451272	-8	462.62	52.6	5.68	Moderately soluble	High	No	No
ZINC000070454761	-8	442.67	57.53	5.15	Moderately soluble	High	No	No
ZINC000095912718	-8	526.57	114.43	2.37	Moderately soluble	High	No	Yes
ZINC000095913855	-8	446.62	75.99	3.57	Moderately soluble	High	Yes	Yes

Table S2. Anti-EBOV inhibition efficiency of the shortlisted compounds using Anti-Ebola.

**Compound**	**Inhibition Efficiency**			
	**Random Forest**		**Support Vector Machine**	
	**pIC50**	**IC_{50} (μM)**	**pIC50**	**IC_{50} (μM)**
**AfroDB**				
ZINC000034518176	5.41	3.86	5.5	3.14
ZINC000095485942	5.53	2.93	4.69	20.51
**NANPDB**				
NANPDB2933 (2-hydroxyseneganolide)	5.15	2	4.3	2
**TCM**				
ZINC000085531689	5.96	1.1	7.05	0.09
ZINC000014089759	5.42	3.83	5.52	3.03
ZINC000085545967	5.46	3.48	5.48	3.35
ZINC000085568136	6	0.99	7.26	0.05
ZINC000095912717	5.61	2.45	5.12	7.62
ZINC000014089743	5.42	3.83	5.52	3.01
ZINC000101564200	5.54	2.9	4.89	12.82
ZINC000085504890	N/A	N/A	N/A	N/A
ZINC000095909661	5.87	1.36	7.65	0.02
ZINC000070454124	5.76	1.75	6.36	0.44
ZINC000085530485	5.81	1.54	5.35	4.45
ZINC000095911418	5.62	2.4	5.88	1.33
ZINC000100822646	5.54	2.9	6.06	0.87
ZINC000100822774	5.67	2.14	6.45	0.36
ZINC000103580868	5.56	2.79	5.23	5.93
ZINC000014587019	5.81	1.56	6.74	0.18

ZINC000033829866	5.49	3.24	5.63	2.36
ZINC000095910238	5.73	1.88	6.89	0.13
ZINC000103580368	5.59	2.56	5	10.12
ZINC000004098631	5.75	1.77	6.68	0.21
ZINC000033833171	5.51	3.07	5.98	1.04
ZINC000070454883	5.52	3.01	5.64	2.3
ZINC000095911414	5.44	3.63	4.68	20.94
ZINC000033833170	5.45	3.56	5.73	1.87
ZINC000070451048	N/A	N/A	N/A	N/A
ZINC000085530490	5.83	1.47	5.38	4.13
ZINC000085592933	5.77	1.71	7.44	0.04
ZINC000095910059	5.45	3.58	5.46	3.45
ZINC000095911347	5.59	2.56	5	10.12
ZINC000095911794	5.57	2.67	6.42	0.38
ZINC000095912227	5.45	3.58	5.78	1.65
ZINC000103554058	5.59	2.56	5	10.12
ZINC000005765422	5.45	3.58	5.76	1.75
ZINC000014587014	5.79	1.63	7.26	0.06
ZINC000014828729	5.02	9.62	4.3	49.77
ZINC000031155872	5.49	3.23	5.34	4.61
ZINC000049888703	5.71	1.97	6.53	0.3
ZINC000070451272	5.63	2.36	5.7	2
ZINC000070454761	5.01	9.77	4.86	13.93
ZINC000095912718	5.52	3.03	5.04	9.06
ZINC000095913855	5.54	2.88	5.66	2.18
**Approved**				
Doramectin	5.74	1.83	7.61	0.02
Ledipasvir	5.81	0	6.66	0
Avermectin B1 (Abamectin)	6.02	0	7.59	0
Elbasvir	5.78	1.67	7.14	0.07
Venetoclax (ABT-199, GDC-0199)	N/A	N/A	N/A	N/A
Revefenacin	0	1000000	0	1000000
Glecaprevir	5.77	1.71	6.44	0.36
Velpatasvir	5.68	2.11	6.73	0.19
Hederacoside C (Kalopanaxsaponin B)	N/A	N/A	N/A	N/A
Paritaprevir (ABT-450)	5.86	1.38	7.17	0.07
Selamectin	5.9	1.25	6.81	0.16
Pibrentasvir (ABT-530)	N/A	N/A	N/A	N/A
Enzastaurin (LY317615)	5.91	1.22	7.22	0.06
Deforolimus (MK-8669, AP23573, MK-8669)	5.57	2.72	6.98	0.1
Nafarelin Acetate	0	0	0	0
Danoprevir (ITMN-191)	5.93	0	7.35	0
Tolvaptan (OPC-41061, Samsca)	5.66	2.17	6.13	0.74
Fangchinoline	5.78	1.68	6.66	0.22
Eltrombopag	5.91	0	6.98	0
Lanatoside C	5.67	0	7.21	0
Nilotinib (AMN-107)	5.71	1.96	6.07	0.85

Ivermectin (Stromectol)	5.91	1.22	6.53	0.3
Glycyrrhizin (Glycyrrhizic Acid)	5.75	0	7.33	0
Sennoside A	5.49	0	6.53	0
Sennoside B	5.49	0	6.53	0
Cepharanthine	5.99	1.03	6.92	0.12
Efonidipine	N/A	N/A	N/A	N/A
Daclatasvir Digydrochloride	5.59	0	5.36	0
BMS-927711 (BHV-3000)	0	1000000	0	1000000
Irinotecan (CPT-11)	0	1000000	0	1000000

Table S3. Interacting residues of the VP40 with nilotinib and the potential lead compounds during molecular dynamics simulations.

**Compound**	**Time (ns)**	**Interacting Residues**	
		**Hydrogen Bonds [Bond Length (Å)]**	**Hydrophobic contacts**
ZINC000034518176	0	Gly139 (3.02)	Phe36, Asn43, Pro47, arg134, Asn136, Arg137, and Leu138
	25	-	Asn43, Gly44, Asp45, Thr123, Arg134, and Asn136
	50	-	Phe36, Asn43, Gly44, Thr123, and Arg134
	75	-	Phe36, Asn43, Gly44, Thr46, Thr121, Thr123, Asn136, and Leu138
	100	-	Gly44, Thr123, and Arg134
ZINC000095485942	0	Thr129 (3.13) and Asn130 (2.97)	Pro131, Gln159, Glu160, Pro165, Leu168, Pro169, and Gln170
	25	-	Gln159, Glu160, Leu163, Pro164, Pro165, Val166, and Phe172
	50	-	Met89, Pro93, Pro164, and Val166
	75	-	Arg2, Gln91, Pro93, Phe161, Val162, Leu163, Pro164, and Pro165
	100	Met1 (2.52 and 2.78)	Arg2, Gln91, Pro93, Ile94, Trp95, Phe161, Val162, Leu163, Pro164, and Pro165
NANPDB2933	0	Gln159 (2.97), Gln167 (3.33 and 3.1), and Gln170 (2.74)	Ala128, Thr129, Pro131, Glu160, Pro165, Val166, and Leu168
	25	His124 (2.91), Val166 (3.03 and 3.28), and Leu168 (3.25)	Met89, Ala128, Thr129, Asn130, Pro131, Glu160, Pro164, Pro165, Gln167, and Phe172
	50	Val166 (2.88 and 3.25), Leu168 (3.2 and 3.34), and Gln170 (2.85)	His124, Ala128, Thr129, Asn130, Pro131, Glu160, Pro164, Pro165, and Phe172

	75	Asn130 (2.85), Val166 (2.82 and 3.18), Leu168 (3.29), and Gln170 (2.97)	Met89, His124, Ala128, Thr129, Pro131, Pro164, Pro165, Gln167, and Phe172
	100	Asn130 (3.05), Val166 (2.79 and 3.31), and Gln170 (2.73)	His124, Ala128, Thr129, Pro131, Val133, Glu160, Leu163, Leu168, and Phe172
ZINC000085531689	0	Asn130 (2.79, 3.16, 3.24), Gln159 (3.02), Glu160 (2.53), and Pro169 (2.63)	Gln167, Leu168, Gln170, and Phe172
	25	Gln167 (2.85)	Lys127, Ala128, Thr129, Leu168, and Gln170
	50	Gln167 (2.66) and Gln170 (3.21)	Leu88, Leu168, and Pro169
	75	Leu168 (2.87) and Gln170 (2.95)	Pro165, Val166, and Gln167
	100	Glu160 (2.95) and Gln167 (3.32)	Phe161, Leu163, Pro164, Pro165, and Val166
ZINC000014089759	0	Pro39 (2.75)	Tyr18, Pro19, Arg21, Asn23, and Lys127
	25	-	Arg3, Val4, Tyr13, Tyr18, Pro19, Asn130, Leu158, and Phe161
	50	-	Arg3, Tyr13, Tyr18, and Pro19
	75	-	Pro11, Glu12, and Tyr13
	100	-	-
ZINC000085568136	0	-	Val42, Asn43, Gly44, Asp45, Gly84, Lys127, Ala128, and Tyr171
	25	Gln170 (3.28)	Val42, Asn43, Gly44, Asp45, His124, Gly126, Lys127, Ala128, and Tyr171
	50	Gln170 (3.23)	Val42, Lys127, Ala128, Thr19, and Tyr171
	75	-	Asn43, Gly44, Gly126, Lys127, Ala128, Thr129, Gln170, and Tyr171
	100	-	Asn43, Gly126, Lys127, Ala128, Thr129, Gln170, and Tyr171
ZINC000095912717	0	Thr129 (2.9), Asn130 (2.94), and Gln170 (2.71)	Lys127, Ala128, Pro131, Gln159, Glu160, Pro164, Pro165, Val166, Gln167, and Leu168
	25	Asn130 (3.01) and Leu168 (3.33)	Pro131, Gln159, Glu160, Leu163, Pro164, Pro165, Val166, Gln167, and Gln170
	50	Met1 (3.13) and Gln159 (2.82)	Glu160, Pro164, Pro165, Val166, Gln167, and Leu168
	75	Leu168 (2.99)	Thr129, Asn130, Pro131, Gln159, Pro165, Val166, and Gln170
	100	Arg3 (2.8)	Asn130, Pro131, Gln159, Glu160, Pro165, Val166, Gln167, and Gln170
Nilotinib	0	-	Tyr13, Tyr18, Pro19, Val20, arg21, Pro39, Glu40, Val42, Lys127
	25	-	Val20, Thr38, Glu40, ser41, Val42, Phe125, and Ala156
	50	-	Pro19, Val20, Thr38, Glu40, Ser41, Val42, Asn43, Phe125, Gly126, Asn130, and Leu132
	75	-	Val20, Arg21, Glu40, and Val42
	100	-	-

Supplementary Figures

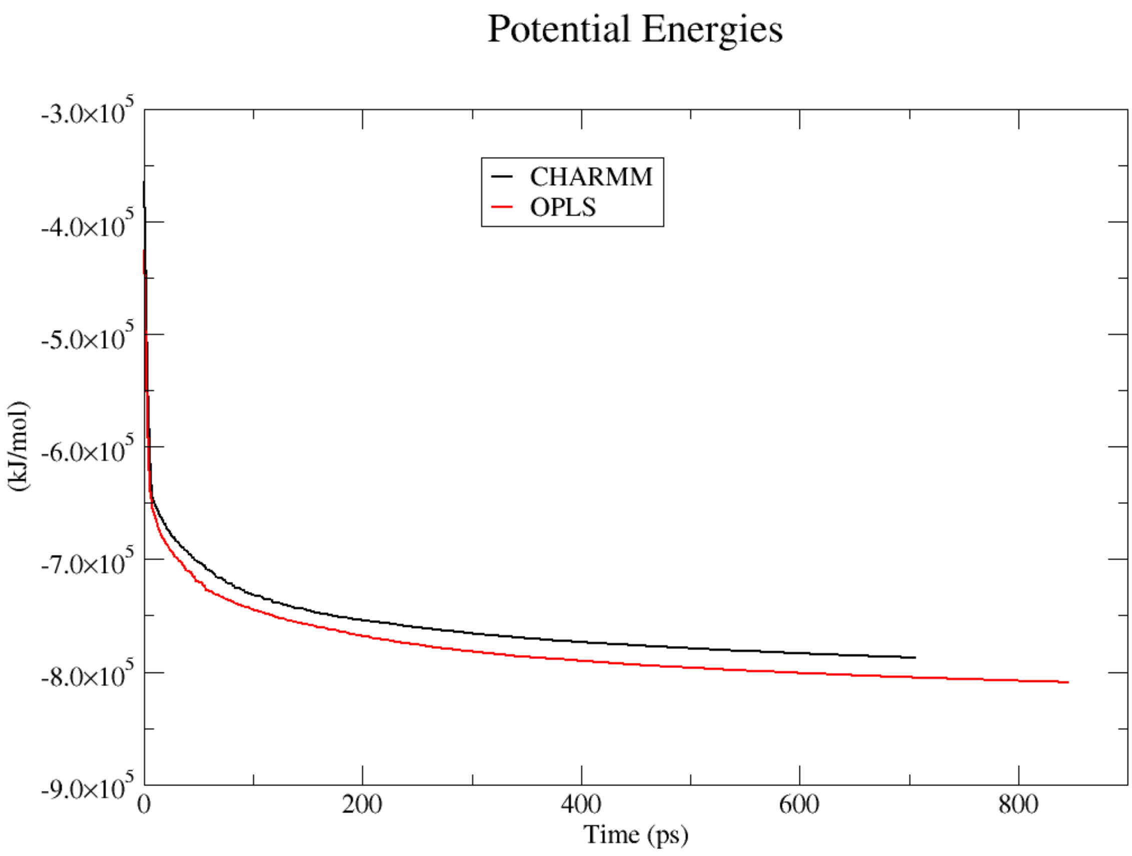

Figure S1. Potential energy plot of the VP40 after subjecting the protein to energy minimization using CHARMM-36 and OPLS force fields.

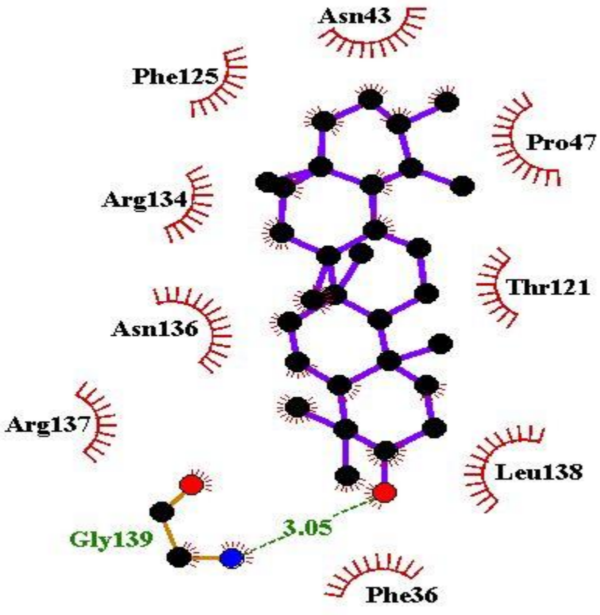
Asn43
Phe125
Pro47
Arg134
Thr121
Asn136
Arg137
Leu138
Gly139
3.05
Phe36

(a)

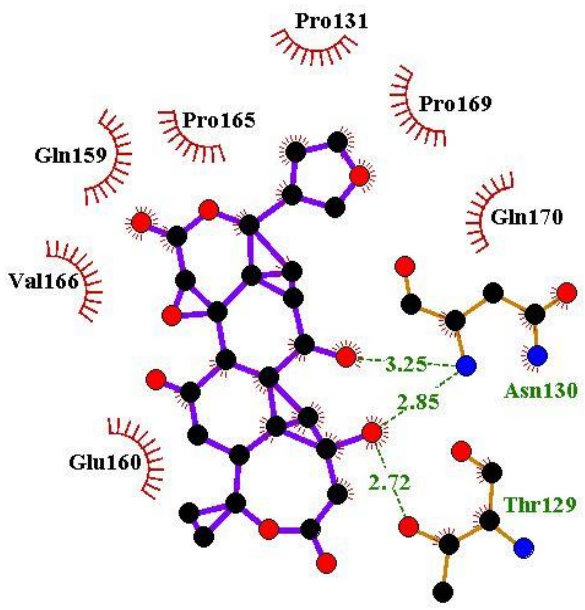
Pro131
Pro169
Pro165
Gln159
Gln170
Val166
3.25
Asn130
2.85
Glu160
2.72
Thr129

(b)

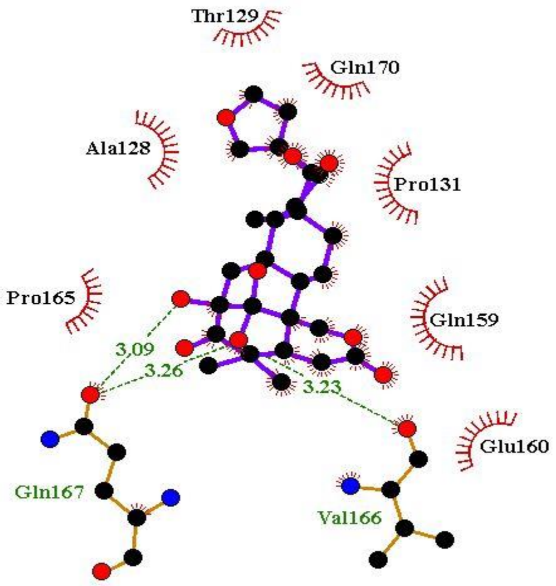
Thr129
Gln170
Ala128
Pro131
Pro165
Gln159
3.09
3.26
3.23
Glu160
Gln167
Val166

(c)

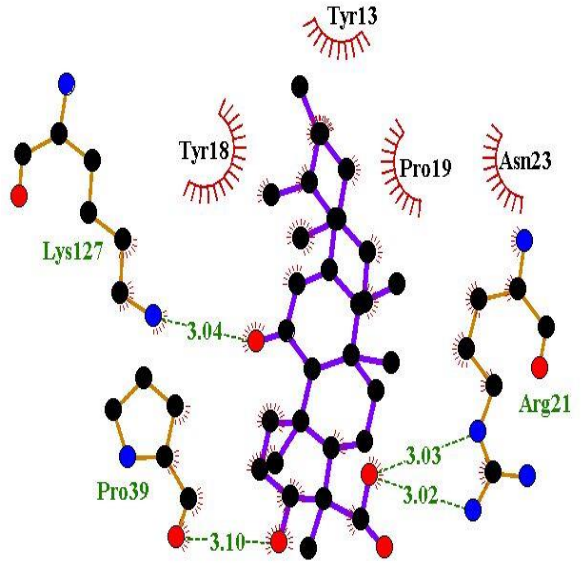
Tyr13
Tyr18
Pro19
Asn23
Lys127
3.04
Arg21
Pro39
3.03
3.02
3.10

(d)

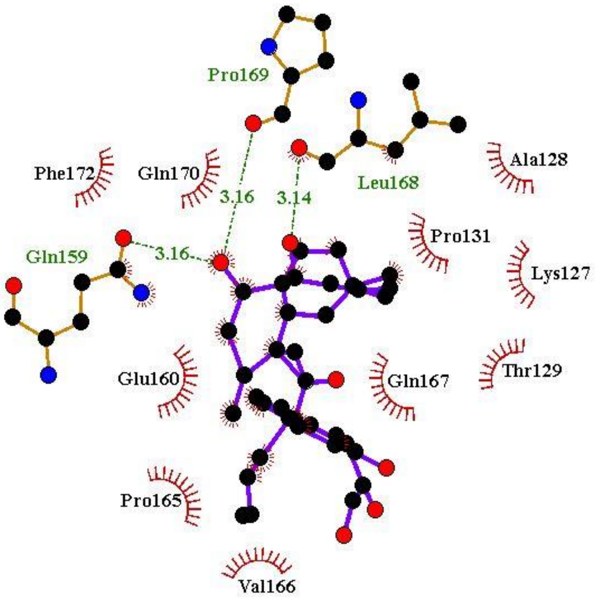
Pro169
Phe172
Gln170
3.16
3.14
Leu168
Ala128
Pro131
Gln159
3.16
Lys127
Glu160
Gln167
Thr129
Pro165
Val166

(**e**)

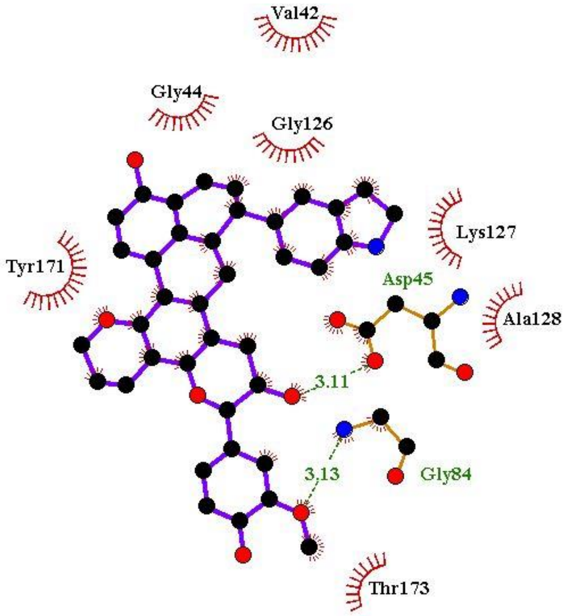
Val42
Gly44
Gly126
Lys127
Tyr171
Asp45
Ala128
3.11
3.13
Gly84
Thr173

(**f**)

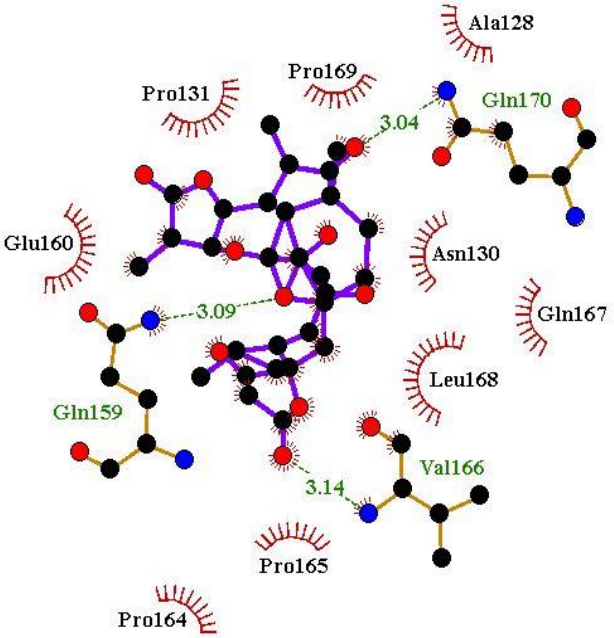
Ala128
Pro131
Pro169
Gln170
3.04
Glu160
Asn130
3.09
Gln167
Leu168
Gln159
Val166
3.14
Pro165
Pro164

(**g**)

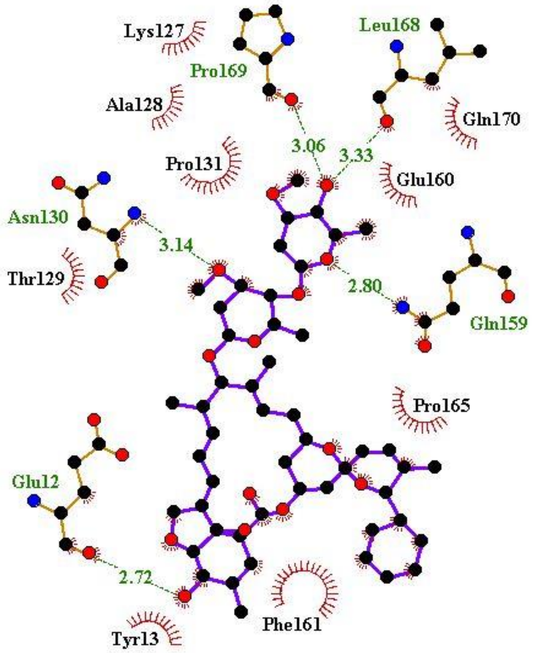
Lys127
Leu168
Pro169
Ala128
Gln170
3.06
3.33
Pro131
Glu160
Asn130
3.14
Thr129
2.80
Gln159
Pro165
Glu12
2.72
Tyr13
Phe161

(**h**)

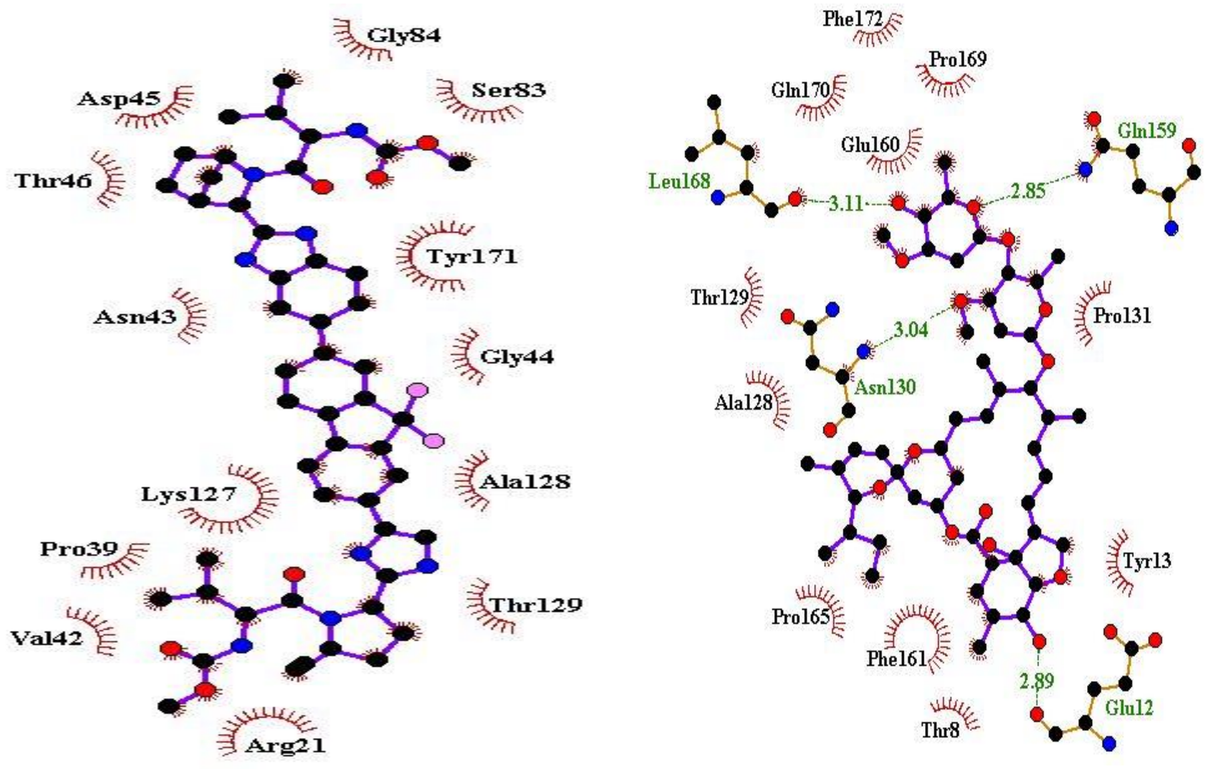

(**i**) (**j**)

Figure S2. Protein-ligand interaction map of VP40 in complex with: (**a**) ZINC000034518176; (**b**) ZINC000095485942; (**c**) NANPDB2933; (**d**) ZINC000014089759; (**e**) ZINC000085545967; (**f**) ZINC000085568136; (**g**) ZINC000095912717; (**h**) doramectin; (**i**) ledipasvir; and (**j**) abamectin. The green dotted lines indicate hydrogen bonds while the red arcs with spikes represent hydrophobic interactions.

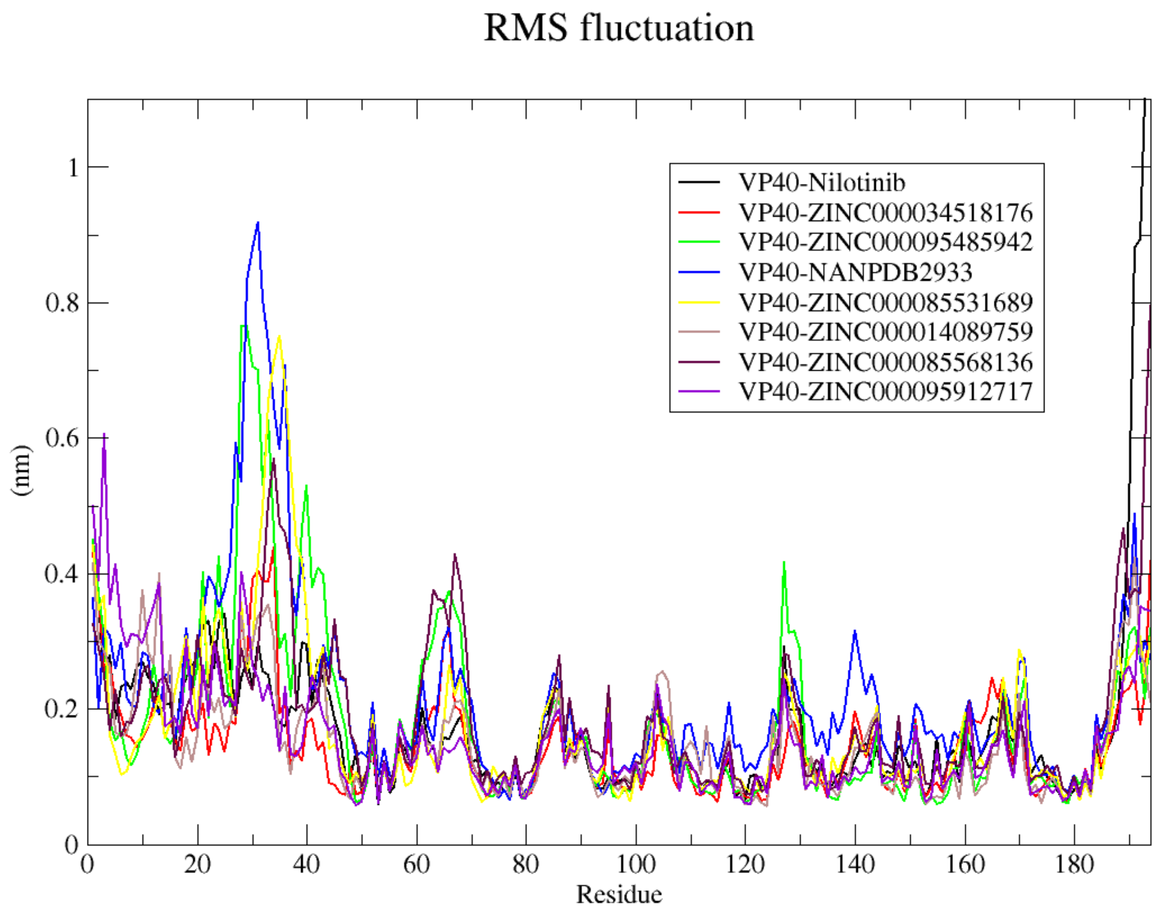

Figure S3. RMSF plot of the EBOV VP40-ligand complexes after 100 ns MD simulations. The VP40-nilotinib, VP40-ZINC000034518176, VP40-ZINC000095485942, VP40-NANPDB2933, VP40-ZINC000085531689, VP40-ZINC000014089759, VP40-ZINC000085568136, and VP40-ZINC000095912717 complexes are colored black, red, green, blue, yellow, brown, maroon, and violet, respectively.

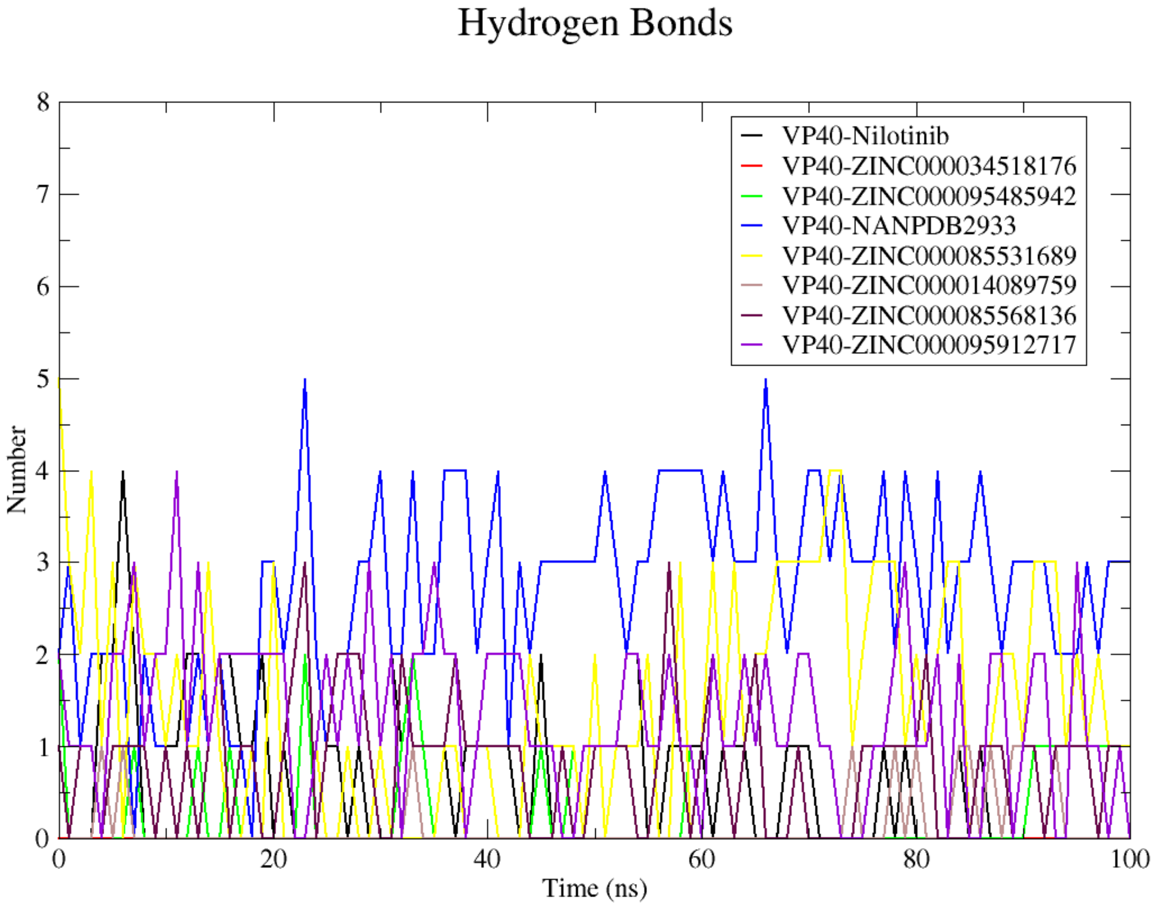

Figure S4. Hydrogen bond analysis plot of the EBOV VP40-ligand complexes after 100 ns MD simulations. The VP40-nilotinib, VP40-ZINC000034518176, VP40-ZINC000095485942, VP40-NANPDB2933, VP40-ZINC000085531689, VP40-ZINC000014089759, VP40-ZINC000085568136, and VP40-ZINC000095912717 complexes are colored black, red, green, blue, yellow, brown, maroon, and violet, respectively.

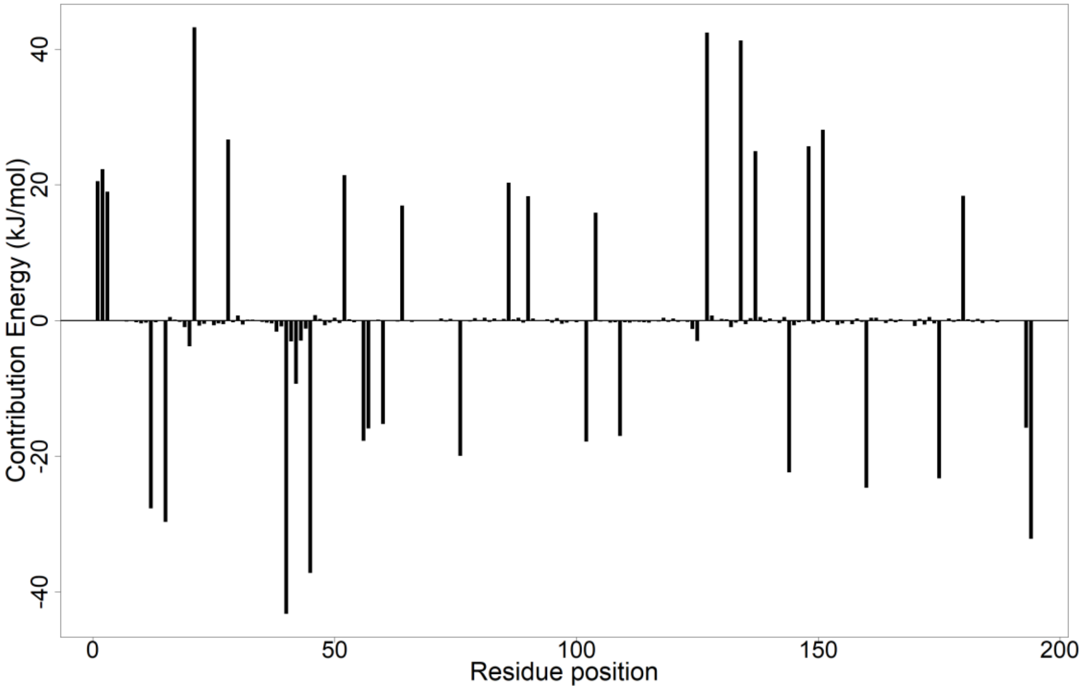

Contribution Energy (kJ/mol)
40
20
0
-20
-40
0
50
100
150
200
Residue position

(**a**)

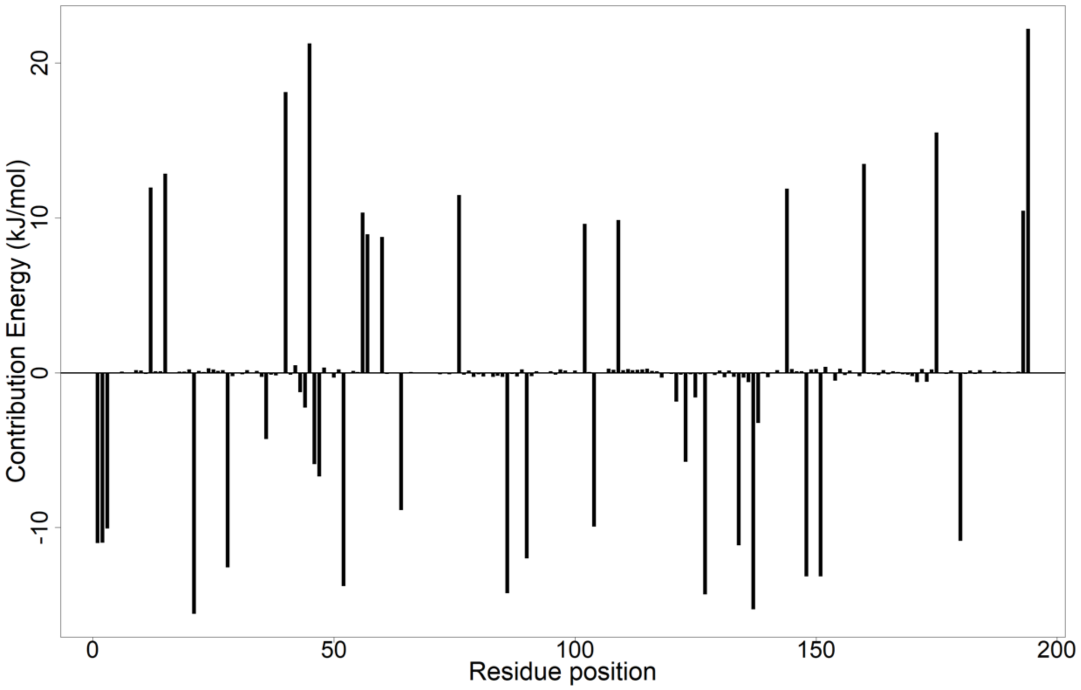

Contribution Energy (kJ/mol)
20
10
0
-10
0
50
100
150
200
Residue position

(**b**)

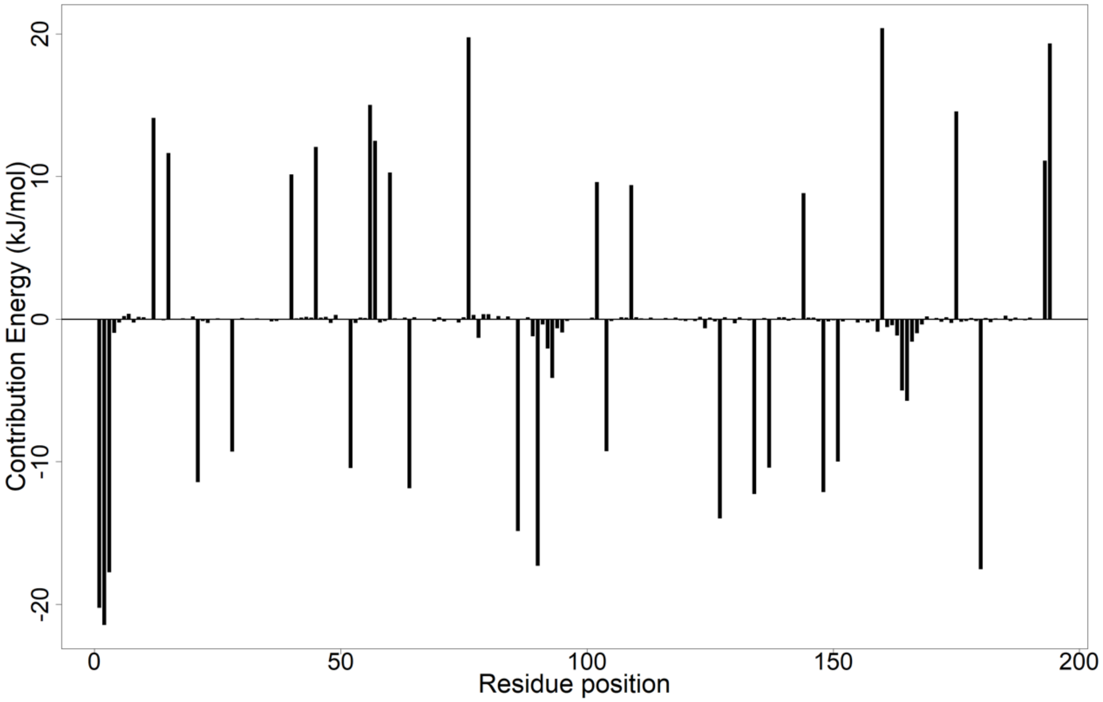

Contribution Energy (kJ/mol)
20
10
0
-10
-20
0
50
100
150
200
Residue position

(c)

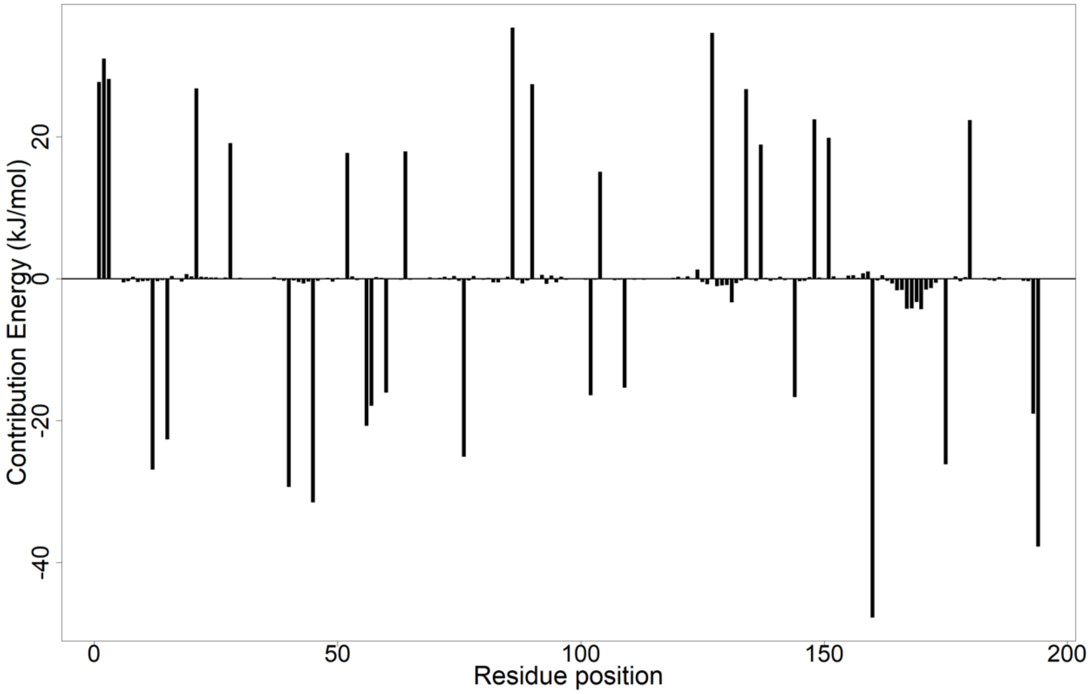

Contribution Energy (kJ/mol)
20
0
-20
-40
0
50
100
150
200
Residue position

(d)

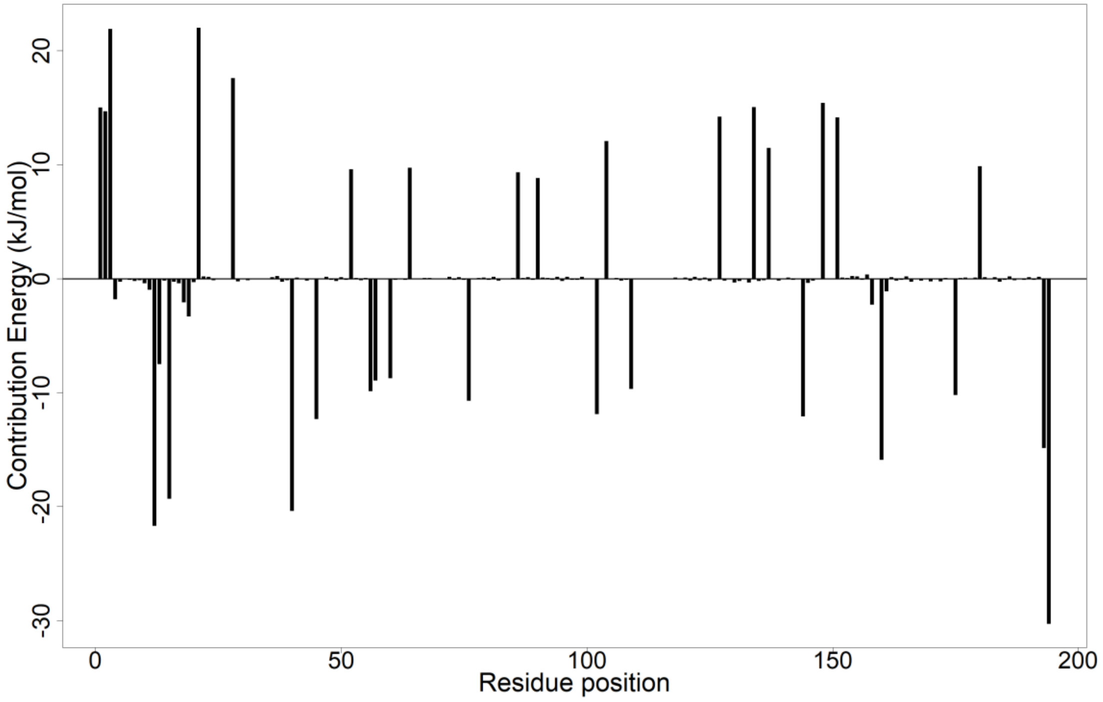
Contribution Energy (kJ/mol)
Residue position
20
10
0
-10
-20
-30
0
50
100
150
200

(**e**)

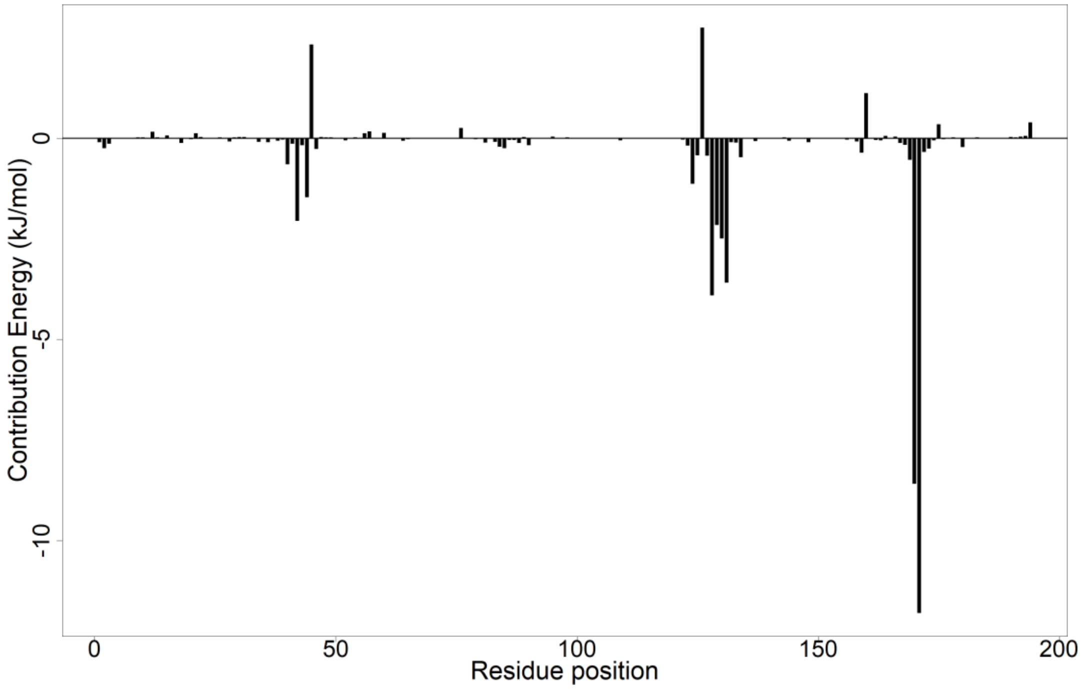
Contribution Energy (kJ/mol)
Residue position
0
-5
-10
0
50
100
150
200

(**f**)

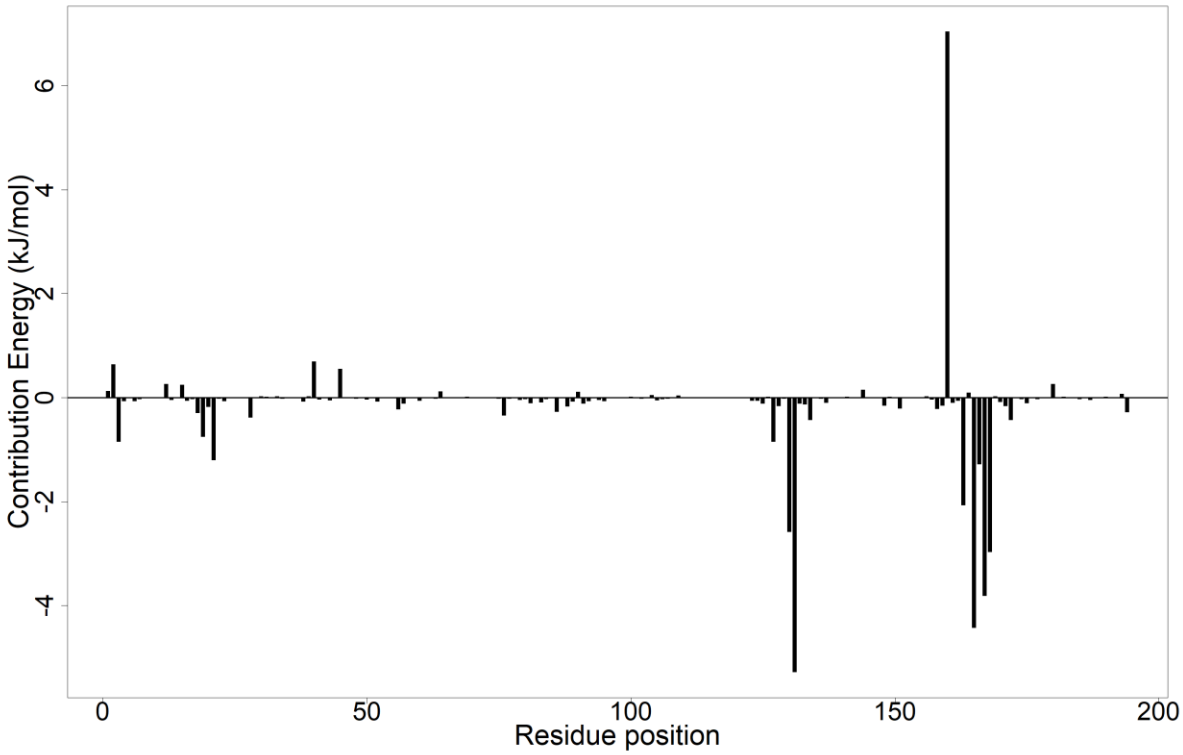

(g)

Figure S5. Per-residue energy decomposition plots of the various complexes showing the energy contributions of each amino acid residue for (a) VP40-nilotinib, (b) VP40-ZINC000034518176, (c) VP40-ZINC000095485942, (d) VP40-ZINC000085531689, (e) VP40-ZINC000014089759, (f) VP40-ZINC000085568136, and (g) VP40-ZINC000095912717 complexes.
